# Supplementary material for: Clinical Role of Upfront F-18 FDG PET/CT in Determining Biopsy Sites for Lung Cancer Diagnosis
Source: Diagnostics (Basel). 2024 Jan 9;14(2):153. doi: 10.3390/diagnostics14020153 (PMC10813933; doi:10.3390/diagnostics14020153)
Supplement: Supplementary file 1 [file diagnostics-14-00153-s001.zip › diagnostics-2792630-supplementary.pdf]

Table S1. Biopsy outcomes: sample adequacy rate and diagnostic accuracy with associated factors

Variables	Adequate biopsy (n = 1265)	Inadequate biopsy (n = 32)	P-value
Sex			0.285
Male	898 (71.0)	26 (81.2)	
Female	367 (29.0)	6 (18.8)	
Age†, years	71.3 ± 10.2	72.2 ± 12.3	0.645
Number of cores obtained†	1.9 ± 1.0	1.9 ± 1.2	0.801
Guidance modality			0.785
CT	368 (29.1)	9 (28.1)	
Cone-beam CT	113 (8.9)	4 (12.5)	
Ultrasound	784 (62.0)	19 (59.4)	
Operator			0.304
Radiologist A	654 (51.7)	20 (62.5)	
Radiologist B	611 (48.3)	12 (37.5)	
Variables	Diagnostic success (n = 1211)	Diagnostic failure (n = 86)	P-value
Sex			0.297
Male	858 (70.9)	66 (76.7)	
Female	353 (29.1)	20 (23.3)	
Age†, years	71.4 ± 10.2	70.7 ± 10.4	0.561
Number of cores obtained†	1.9 ± 1.0	1.9 ± 1.2	0.704
Guidance modality			0.072
CT	347 (28.7)	30 (34.9)	
Cone-beam CT	105 (8.7)	12 (14.0)	
Ultrasound	759 (62.7)	44 (51.2)	
Operator			0.686
Radiologist A	627 (51.8)	47 (54.7)	
Radiologist B	584 (48.2)	39 (45.3)	

Note—Except where otherwise indicated, data are number with percentage in parentheses.

† Continuous variables were presented as mean ± standard deviation variables.

Disclaimer/Publisher's Note: The statements, opinions and data contained in all publications are solely those of the individual author(s) and contributor(s) and not of MDPI and/or the editor(s). MDPI and/or the editor(s) disclaim responsibility for any injury to people or property resulting from any ideas, methods, instructions or products referred to in the content.
